# Generation of Reactive Oxygen Species during Apoptosis Induced by DNA-Damaging Agents and/or Histone Deacetylase Inhibitors

**DOI:** 10.1155/2011/253529

**Published:** 2011-09-21

**Authors:** Barbora Brodská, Aleš Holoubek

**Affiliations:** Institute of Hematology and Blood Transfusion, U Nemocnice 1, 12820 Prague 2, Czech Republic

## Abstract

Reactive oxygen species play an important role in the process of apoptosis in many cell types. In this paper, we analyzed the role of ROS in DNA-damaging agents (actinomycin D or decitabine), which induced apoptosis of leukemia cell line CML-T1 and normal peripheral blood lymphocytes (PBL). The possibility of synergism with histone deacetylase inhibitors butyrate or SAHA is also reported. We found that in cancer cell line, ROS production significantly contributed to apoptosis triggering, while in normal lymphocytes treated by cytostatic or cytotoxic drugs, necrosis as well as apoptosis occurred and large heterogeneity of ROS production was measured. Combined treatment with histone deacetylase inhibitor did not potentiate actinomycin D action, whereas combination of decitabine and SAHA brought synergistic ROS generation and apoptotic features in CML cell line. Appropriate decrease of cell viability indicated promising therapeutic potential of this combination in CML, but side effects on normal PBL should be taken into attention.

## 1. Introduction

Increased caspase-dependent apoptosis, reactive oxygen species (ROS) generation and mitochondrial damage are phenomena, which can be frequently observed altogether in cells subjected to anticancer drugs treatment, that is, accumulation of ROS inside the cell often signalizes apoptosis or terminal differentiation [1]. On the other hand, interleukins-(IL-7- and IL-3-) induced ROS generation provides cell survival [2, 3]. Among the agents upregulating ROS, we can find natural compounds (EGCG, curcumin, or garlic [4–6]), anti-inflammatory agents (parthenolide, quercetin [7, 8]), anticancer chemical drugs (paclitaxel, cisplatin, doxorubicin [9–11]), and even some antioxidants (e.g., melatonin [12]). Some of the ROS inductions correlate with apoptosis [13], other occur as independent phenomena [14]. In many cases, potentiation of another drug action or sensitization of resisting cells is induced by ROS generation [4, 15–18]. 

Along with ROS generation, DNA damage has usually been observed during the process of cell death. These two phenomena, the increase of ROS level and DNA damage, can be found either independent or one being caused by the other one. Actinomycin D (Dactinomycin, actD) causes breaks in both ds and ssDNA, and cells treated by actinomycin D are reported to be more sensitive to subsequent treatment (TRAIL, TNF-alpha) because of elevating reactive oxygen species concentration [18, 19]. DNA-damage caused by high-concentration of 5-aza-2′deoxycytidine (decitabine, DAC) was reported to be accompanied by caspase-independent ROS generation in myeloma cells [20] as well as by ROS production-dependent apoptosis in p53-mutant leukemic T-cells [21]. No effect on ROS production in normal peripheral blood lymphocytes was detected [22]. At low concentrations, (up to 1 *μ*M) DAC acts as S-phase-specific epigenetic agent (DNA-methyltransferase inhibitor) causing *de novo* DNA hypermethylation and silencing of transcription process. This fact is now widely exploited in new therapeutic strategies [23, 24]. Nevertheless, our experiments are focused on the DNA-damaging effect of DAC occurring at high concentration treatment. Both of these drugs, actD and DAC, are able to induce p53-dependent, mitochondria uncoupling way of apoptosis in leukemia cell line CML-T1, but the apoptosis is induced also in normal lymphocytes [22, 25].

Butyrate (BUT, in form of sodium butyrate), a short-chain fatty acid and natural histone deacetylase inhibitor, is known to induce terminal cell differentiation in HL-60 cells [26]. Physiological concentrations of butyrate induce ROS that transiently alter intracellular redox balance of intestinal cells [27], preincubation by butyrate protects colonocytes against H_2_O_2_-induced damage [28]. In normal peripheral blood lymphocytes, butyrate induces apoptosis, which is partly mediated by ROS [29]. Another histone deacetylase inhibitor, suberoylanilide hydroxamic acid (Vorinostat, SAHA), increased reactive oxygen species levels in gastrointestinal tumor cells [30] as well as in leukemia [31] or small cell lung cancer cells [32]. These facts together with relatively high resistance of normal cells to SAHA treatment [33, 34] drift this drug to the forefront in anticancer research.

In this paper, we analyze the role of ROS in apoptosis of leukemia cell line CML-T1 and normal peripheral blood lymphocytes (PBL) induced by DNA-damaging agents, actD or DAC, and by histone deacetylase inhibitor, BUT or SAHA. Comparison with effects induced by drug combinations is also reported.

## 2. Material and Methods

### 2.1. Cell Culture

Human peripheral blood lymphocytes of healthy donors were isolated from buffy coats using density gradient centrifugation on Histopaque 1077 (Sigma-Aldrich Corporation, St. Louis, MO, USA) at 500 g and 20°C for 25 min. Histopaque-concentrated layer was resuspended in RPMI 1640 (Biochrom AG, Germany) for 45 min and monocytes were depleted by harvesting nonadherent cells. Lymphocytes were resuspended at a density of 1 × 10^6^ cells/mL in RPMI 1640 medium (10% FCS, 1% penicillin + streptomycin). CML-T1 cells were cultured in RPMI 1640 at starting density of 5 × 10^5^ cells/mL. Actinomycin D, Sodium Butyrate (both from Sigma-Aldrich), SAHA (Cayman Chemical Company, Ann Arbor, MI, USA), and Decitabine (Sigma-Aldrich) were added separately or in combinations (concurrently) for time periods from 0 up to 48 h (CML-T1) or 72 h (PBL) at 37°C in 5% CO_2_. Concentrations used for each cell type are indicated in Table 1.

### 2.2. Flow Cytometry

Cell viability (monitored by propidium iodide, PI), generation of reactive oxygen species (ROS, observed by dichlorodihydrofluorescein diacetate, H_2_DCFDA), and mitochondrial membrane potential (MMP, measured by MitoTracker Red, MTR) were investigated by flow cytometry. All fluorescent probes were purchased from Invitrogen (Carlsbad, CA, USA). Treated cells (2 × 10^6^ for CML, 1 × 10^7^ for PBMC) were harvested, washed with PBS, and suspended in 1 mL PBS. H_2_DCFDA or MTR was added to final concentration of 10 *μ*M (H_2_DCFDA) or 40 nM (MTR) for 30 min in 5% CO_2_ at 37°C. After washing and resuspending the cells in PBS, samples (50 000 events/sample) were analyzed on a FACSCalibur Flow Cytometer (BD Biosciences, San Jose, CA, USA). Ground level of ROS production was established as H_2_DCFDA fluorescence intensity reached by 50% of untreated cells. Region of viable cells was defined as such area in scattergram, which was stable for all cell samples, and the shift of H_2_DCFDA spectra was quantified as a proportion of cells reaching fluorescence intensity above the ground level. In diagrams arithmetic means of at least four times repeated experiments were plotted with SEM error bars. Significance levels (*P* values of ANOVA analyses) were determined using InStat Software (GraphPad Software). A *P* value of 0,05 or lower was considered to be a statistically significant difference between the groups compared. The extent of mitochondria staining by MitoTracker Red redistribution probe was investigated in order to observe mitochondrial membrane potential changes during cell exposure to different drugs. Fluorescence intensity threshold of polarized mitochondria was defined as a minimal intensity reached by MTR-stained untreated cells. Proportion of cells with polarized mitochondria (i.e., cells reaching the fluorescence intensity threshold) from each sample was calculated and used as a marker of apoptosis. For PI-staining, 2 *μ*L of 250 *μ*g/mL propidium iodide stock solution were added to 0,5 mL of PBS-washed cell suspension just before measurement. Fraction of living cells was then defined as an amount of PI-negative stained cells.

### 2.3. Immunoblotting

Cells were cultured for intervals up to 44 h (CML-T1) or 72 h (PBL) in the absence or presence of DNA-damaging agent (actinomycin D or decitabine) and/or histone deacetylase inhibitor (sodium butyrate or SAHA), harvested from cultures, washed in PBS, and then boiled for 5 min in 200 *μ*l of Laemmli buffer. After centrifugation at 200,000 g and 4°C for 3 h, the supernatants were subjected to 12% SDS PAGE electrophoresis and the separated proteins were semidry blotted onto PVDF transfer membranes (Amersham Biosciences, Piscataway, NJ, USA). To measure PARP, *β*-actin, and capase 3 expression levels, blots were developed as described previously [25]. Briefly, membranes were blocked with 5% nonfat dried milk in PBS/Tween and primary antibodies were added overnight with a final dilution range of 1 : 500 to 1 : 2000. After washing, Pierce's HRP-conjugated secondary antibodies (1 : 20,000–1 : 100,000) were added for 2 h (Pierce Biotechnology, Inc. Rockford, IL, USA). Finally the blots were visualized using the ECL Plus chemiluminescent substrate and ECL HyperfilmTM (both from Amersham). Signals were quantified using the Power Look III scanner (UMAX Technologies, Inc. Dallas, TX, USA) and image analyzer Software AIDA 1D. *β*-actin expression served as a loading control. Primary rabbit polyclonal anti-PARP and mouse monoclonal anti-caspase 3 were from Santa Cruz Biotechnology, Inc. (Santa Cruz, CA, USA), mouse monoclonal anti-*β*-actin was originated from Sigma-Aldrich.

## 3. Results

### 3.1. Cell Viability

**Figure 1:**
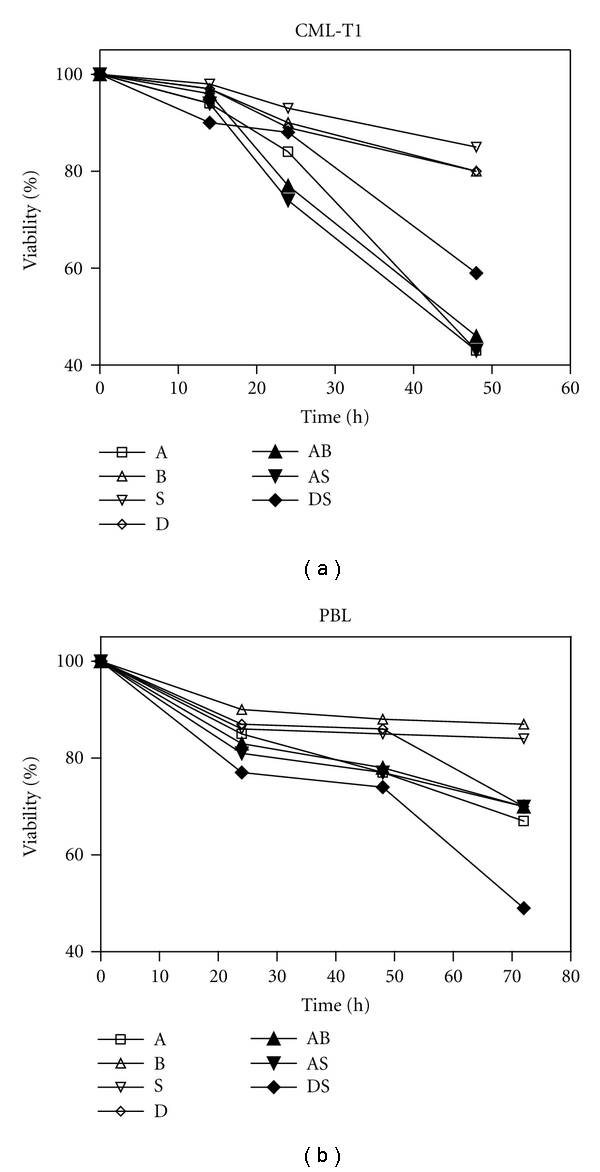
Cell viability assessment in CML cells (a) and PBL (b) treated by actinomycin D (A), sodium butyrate (B), SAHA (S), decitabine (D) or their combination (AB, AS, DS).

Viability of the CML-T1 (CML) cells and normal peripheral blood lymphocytes (PBL) after treatment with DNA-damaging drugs (actD or DAC), histone deacetylase inhibitors (BUT or SAHA), and their combinations was assessed (Figure 1).

Concentrations of particular drugs were experimentally determined to reach a similar effect for both cell types. Therefore, actD and SAHA concentrations for PBL treatment were doubled in comparison with concentrations used for CML, while BUT and DAC were identical for CML and PBL. Even these corrections did not erase the difference between actD action on CML and PBL. In CML, actD caused more than 50% viability drop, and addition of histone deacetylase inhibitor (mostly non-toxic when applied alone) shortened the phase of relative cell resistance from 24 h to 16 h. Only moderate DAC individual action was strongly potentiated by SAHA addition, namely, at 48 h exposure. ActD was substantially less toxic for PBL and HDACi addition had almost no effect on PBL viability. However, SAHA potentiation of decitabine toxicity was observed in PBL too. In conclusion, viability curves of combined treatment indicate the possibility of DAC + SAHA synergism in CML as well as in PBL.

### 3.2. ROS Generation

**Figure 2:**
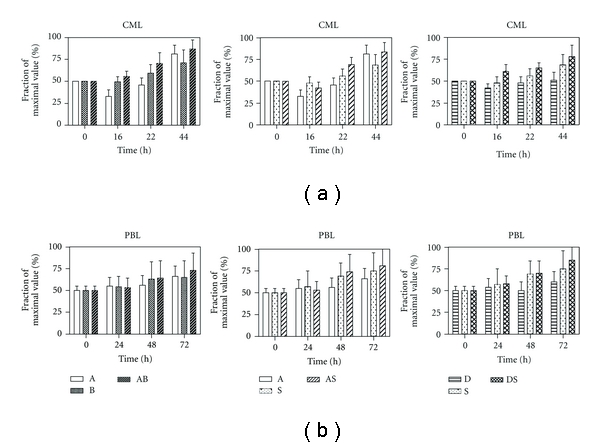
ROS production induced by actinomycin D (A), sodium butyrate (B), SAHA (S), decitabine (D) or their combination (AB, AS, DS) in CML cells (a) or PBL (b) during 44 h (CML) or 72 h (PBL) treatment.

Generation of reactive oxygen species in cells was monitored during the treatment with different drugs or their combinations (Figure 2). *P* values of individual drugs action statistics and significance of combined treatments effects are summarized in Table 2.

In CML cells, ROS production was lowered at initial 16 h and reached its original level after 24 h of actD action, whilst ROS concentration was substantially increased as compared with control cells 48 h after actD addition. Transient drop of ROS concentration was observed also in cells treated by DAC, but ensuing increase of ROS production was not large enough to reach the initial concentration. BUT or SAHA treatment brought gradual increase of ROS production in CML. Combination of actD with any histone deacetylase inhibitor resulted mostly in sligtly enhanced ROS production in comparison with HDACi only-induced ROS, irrespectively to relevant ROS level generated by actD alone. Similarly, DAC reduced ROS level when it was used as single agent, but DAC + SAHA combination enhanced ROS generation in comparison with action of SAHA alone. 

In PBL, actD or DAC treatment caused only small fluctuations of ROS production over the control levels. Histone deacetylase inhibitors induced ROS accumulation alike in CML cells, but the data sets obtained from different buffy coats were largely dispersed. Almost no potentiating effects were detected when combinations with actD were tested. Contrary to CML, even combination of DAC with SAHA was not synergistic in PBL from the ROS production aspect.

### 3.3. Features of Apoptosis

**Figure 3:**
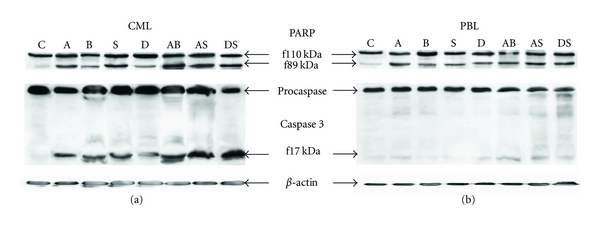
Western blots showing PARP fragmentation and caspase 3 activation during 24 h treatment of CML cells (a) or PBL (b). Cells were treated by actinomycin D (A), sodium butyrate (B), SAHA (S), decitabine (D), or their combination (AB, AS, DS). Symbol C denotes untreated cells. *β*-actin expression is showed as a loading control.

PARP fragmentation and caspase 3 activation were monitored along with ROS production measurements; representative immunoblots after 24 h treatment are shown in Figure 3. In accordance with viability measurements, actD caused PARP fragmentation referring considerable cell injury, while only moderate PARP fragmentation was induced by DAC in both types of cells. Effect of histone deacetylase inhibitors differed by HDACi, while significant PARP fragmentation is caused by SAHA, after sodium butyrate exposure PARP fragmentation was only weak. Herein, some potentiating effect brought all the combinations tested. In contrast to these results, large caspase 3 activation was only detected for CML cells. According to the PARP fragmentation characteristics, significant executive caspase activation was observed mainly in CML cells treated by actD—either alone or in combinations—and also in experiments with DAC + SAHA combination. 

**Figure 4:**
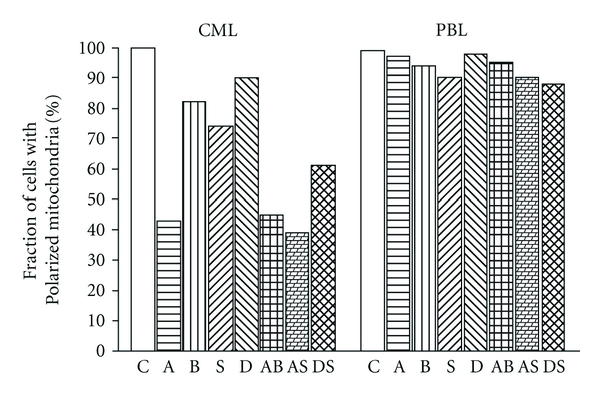
Mitochondrial membrane depolarization after 24 h treatment of CML cells or PBL. Cells were treated by actinomycin D (A), sodium butyrate (B), SAHA (S), decitabine (D), or their combination (AB, AS, DS). Symbol C denotes untreated cells.

Figure 4 shows changes of another marker of intrinsic apoptosis pathway, mitochondrial membrane potential, in response to 24 h treatment by individual drugs or by their combinations. Consistently with foregoing results, no potentiation of mitochondrial membrane depolarization caused by HDACi in combination with actD was possible to observe in CML-T1 cells. Only weak effect had all the drugs and their combinations on mitochondrial membrane potential of normal PBL after 24 h of action.

## 4. Discussion

Apoptosis is a physiological process in normal cells and an invalid apoptosis pathway has often been one of the main hallmarks of cancer cells. Therefore, large attention is paid to processes leading to changes in apoptosis. ROS formation frequently occurs in the course of apoptotic process and its contribution to programmed cell death is unquestionable. Large scale of cytotoxic and cytostatic drugs offers many ways to induce apoptosis or terminal differentiation in tumor cells. However, most of them influence, to some extent, also the viability of normal cells, and this feature represents unwanted side effect of cancer therapy. Combined action of more drugs applied together may minimalize these unadvisable phenomena by targeting processes specific for proliferating (cancer) cells.

DNA-damaging agents, actD and highly concentrated DAC, belong to the group of very effective, but robust drugs, whose side effects are significant. Histone deacetylase inhibitors, sodium butyrate and SAHA, are epigenetic drugs causing mostly terminal differentiation and as such they have only small effect on differentiated, quiescent cells. Increasing focus on epigenetic mechanism of DAC action when used in low concentrations gives reason for our intention of further study of DAC and SAHA combined epigenetic effect.

We investigated the role of ROS in process of cell death induced by DNA-damaging drugs or by histone deacetylase inhibitors (HDACis), or by combination of these two types of drugs. Leukemia cell line CML-T1 (CML) and normal peripheral blood lymphocytes of healthy donors (PBL) were studied to compare the effect on proliferating cells with functionless apoptotic signaling and on quiescent cells with intact cell death machinery.

At first, viability of CML cells treated by drugs in clinically used concentrations for time intervals up to 48 h was tested, and relevant drug concentrations were constituted for time frame up to 72 h to reach comparable effects on viability of PBL. While 8 *μ*M DAC and 2 mM BUT caused similar effect in both cell types, 1 *μ*M SAHA and 5 nM actD had to be increased two times to induce similar viability drop in PBL. Combined treatment with HDACis did not enhance actD-induced cell death. In parallel, actD-induced markers of apoptosis, for example, p53 stabilization, Puma induction, or mitochondria depolarization, were not potentiated by the HDACi presence. From this point of view, there was no improvement of therapeutic effect of actD alone on CML but neither side effect was worsened by the combination. Viability of CML is substantially decreased using SAHA as potentiating agent for DAC treatment. Unfortunately, SAHA potentiates DAC action also in lymphocytes. Here, we suppose the viability drop to be caused predominantly by nonapoptotic way of the cell death [22]. Considering different concentrations of SAHA used for CML and PBL, we tested the effect of 1 *μ*M SAHA on PBL. Indeed, 1 *μ*M SAHA had no potential to substantially increase the effect of DAC on viability of normal PBL (data not shown). 

As it was described in Introduction, all the drugs tested are reported to increase the ROS level in some type of cells, frequently contributing to apoptosis induction. In our cells, extensive ROS production was detected after HDACi treatment in both cell types, but the data sets obtained from different lymphocytes donors were much more dispersed than those from experiments with CML. While moderate continual increase of ROS production was measured in actinomycin D-treated PBL too, longer time of action was necessary to induce the increase of ROS generation by actD in CML, and this ROS induction was preceded by significant ROS level decrease in 16 h interval. Appropriate response delay was observed also in the viability curve of actD-treated CML, and this delay did not appear if the cells were treated by actD in combination with arbitrary histone deacetylase inhibitor. Moreover, actD + HDACi combined treatment manifested slightly higher ROS production than HDACi alone treatment did, irrespective to actD contribution and the time of action. A significant difference between CML and PBL we can find also in DAC action. While absence of any changes in DAC-treated PBL is reflected by no effect on SAHA-induced ROS production in DAC + SAHA combined treatment, transient decrease of ROS in DAC-treated cells and extensive ROS generation induced by DAC + SAHA are observed in CML.

The extent of poly (ADP-ribose) polymerase (PARP) cleavage serves as a measure of apoptotic effect in cells. In our cells, PARP fragmentation reflects the viability trends for actD, BUT, DAC, and all combined treatments, but larger PARP fragmentation occurs also in SAHA-treated cells. Accordingly, with the extent of PARP cleavage, extensive caspase 3 activation was observed in CML treated by actD and all combinations tested; large caspase 3 activation was monitored also in SAHA treated cells, but only moderate effect was detected when BUT or DAC was used to treat the cells. Surprisingly, only poor or no active caspase 3 fragments were detected in PBL samples by immunoblotting. We detected slight caspase 3 activation in treated PBL by other methods—fluorimetric or luminometric measurement of covalently labelled caspase inhibitor cleavage. But while the amount of active executive caspases continually increased in CML during the 48 h after drug addition, in PBL transient caspase 3 activation in 24 h was followed by subsequent decrease under the control level (data not shown). We conclude, that in proliferating CML cells, all drugs and combinations tested induced mitochondrial way of apoptosis and that ROS production significantly assisted in apoptosis induction. In PBL, large viability drop was accompanied by appropriate PARP fragmentation, but any significant ROS production changes and executive caspases activation were not detected during the treatment. This fact confirmed the thesis that PARP fragmentation was not always dependent on caspase 3 activation [35] and that other factors were involved in triggering of this type of PARP cleavage. Therefore, we can conclude that necrosis as well as apoptosis took part in lymphocytes viability decrease, and this variability is accompanied also by large deviations in amount of ROS produced in the treated PBL.

## 5. Conclusion

In our study, we investigated the role of reactive oxygen species production during the anticancer drugs treatment of two types of cells. Different DNA-damaging drugs and histone deacetylase inhibitors were used to treat cancer or normal cells. We found that in cancer cell line CML-T1, ROS production significantly contributed to apoptosis triggering, while in normal lymphocytes treated by cytostatic or cytotoxic drugs, necrosis as well as apoptosis occurred and large heterogeneity of ROS production was measured. Combined treatment with histone deacetylase inhibitor did not potentiate actinomycin D action, whereas combination of decitabine and SAHA brought synergistic ROS generation and apoptotic features in CML cell line. Appropriate decrease of cell viability indicated promising therapeutic potential of this combination in CML. However, although there was no synergistic effect in ROS production, the viability drop of PBL treated by decitabine was also augmented by combination with SAHA denoting that cell death was induced also in PBL. Further study on both drugs concentrations optimization is therefore needed to enhance the possibility to use this combination in cancer therapy.

## Figures and Tables

**Table 1:** Concentrations of drugs used for treatment of each cell type.

	Actinomycin D	Sodium butyrate	SAHA	Decitabine
CML-T1	5 nM	2 mM	1 *μ*M	8 *μ*M
PBL	10 nM	2 mM	2 *μ*M	8 *μ*M

**Table:** (a)

Cell type	CML-T1	PBL
Drug		
Actinomycin D	<0,0001	0,12
Sodium butyrate	0,03	0,38
SAHA	0,003	0,08
Decitabine	0,16	0,34

**Table:** (b)

Cell type	CML-T1	PBL
Combination		

ActD + BUT	0,03	0,74
ActD + SAHA	0,04	0,74
DAC + SAHA	0,003	0,52
